# The Inhibitory Effect of Artesunate on Excessive Endoplasmic Reticulum Stress Alleviates Experimental Colitis in Mice

**DOI:** 10.3389/fphar.2021.629798

**Published:** 2021-03-09

**Authors:** Shaojie Yin, Liuhui Li, Ya Tao, Jie Yu, Simin Wei, Mingjiang Liu, Jingui Li

**Affiliations:** ^1^ College of Veterinary Medicine, Yangzhou University, Yangzhou, China; ^2^ Jiangsu Co-innovation Center for Prevention and Control of Important Animal Infectious Diseases and Zoonoses, Yangzhou, China; ^3^ Joint International Research Laboratory of Agriculture and Agri-Product Safety, the Ministry of Education of China, Yangzhou University, Yangzhou, China; ^4^ The Affiliated Hospital of Yangzhou University, Yangzhou University, Yangzhou, China

**Keywords:** artesunate, ulcerative colitis, colitis, endoplasmic reticulum stress, inflammation, Intestinal barrier

## Abstract

Endoplasmic reticulum (ER) stress may contribute to the pathogenesis and perpetuation of ulcerative colitis (UC). Previous studies have shown artesuante (ARS) has the protective effect on experimental UC. Therefore, it can be assumed that ARS can regulate ER stress and its related reactions. Dextran sulfate sodium (DSS) induced UC model in mice was used to testify this hypothesis. The results clearly showed that DSS exposure caused excessive ER stress evidenced by a markedly increase of GRP78 and CHOP expression, and then activated the ER stress sensors PERK, IRE1, ATF6 and their respective signaling pathways, followed by upregulated caspases12 and lowered Bcl-2/Bax ratio. However, ARS treatment significantly inhibited the occurrence of ER stress via preventing the activation of PERK-eIF2α-ATF4-CHOP and IRE1α-XBP1 signaling pathways, concurrently ER-stress-associated apoptosis in colon tissues. Moreover, ARS treatment remarkably inhibited the activation of NF-κB and the expression levels of pro-inflammatory cytokines, improved the clinical and histopathological alterations as well as maintained the expression of claudin-1 and Muc2 in mucosal layer of colon. Notably, the classic ER stress inhibitor 4-phenyhlbutyric acid enhanced the beneficial effects of ARS; in contrast, the ER stress inducer 2-deoxy-d-glucose substantially abrogated the above-mentioned effects, uncovering the involvement of ER stress in the response. These findings indicated the protection of ARS on UC is associated with its suppressing excessive ER stress mediated intestinal barrier damage and inflammatory response. This study provides a novel aspect to understand the mechanism of ARS against UC.

## 1 Introduction

Ulcerative colitis (UC), an idiopathic relapsing inflammatory disease, is one main subtype of inflammatory bowel disease (IBD) (Ren et al., 2019). Continuous and diffuse inflammation in UC starts in the rectum and generally extends proximally through the entire colon, as well, the inflammation is typically restricted to the mucosal surface (Ordás et al., 2012; Feuerstein et al., 2019). The etiology and pathogenesis of UC remains unclear and complicated involving a combination of multi-factors including genetic predisposition, environment factors, epithelial barrier defects and dysregulated immune responses (Ordás et al., 2012; Wehkamp et al., 2016). It has been demonstrated that abnormal immune responses and intestinal barrier dysfunction may be key players in the progression of UC (Camilleri et al., 2012; Chen and Sundrud 2016). Maintaining cellular homeostasis is crucial for normal immune function and intestinal barrier integrity (Okumura and Takeda 2018; Sharma and Rudra 2018). When cells are exposed to extra- or intracellular stress which disrupt cellular homeostasis, stress-signaling pathways play an important role in rebalancing the biochemical processes. However, these signaling pathways also engage in establishing dysfunctional states associated with stress exposure and the development of diseases (Hotamisligil and Davis 2016).

Endoplasmic reticulum (ER) is an essential intracellular dynamic organelle mediating the synthesis and export of proteins and glycoproteins and is highly sensitive to disturbances of homeostasis (Senft and Ronai 2015; Ivanova and Orekhov 2016). Multiple factors can influence the homeostasis and induce accumulation of unfolded and misfolded proteins in the ER lumen, which is termed ER stress (Cao 2015). A major response to ER stress in mammalian cells is the activation of unfolded protein response (UPR) signaling initiated by three protein sensors on the ER membrane including protein kinase RNA-like endoplasmic reticulum kinase (PERK), inositol-requiring protein 1 (IRE1) and activating transcription factor 6 (ATF6), which respectively leading to the activation of three signaling pathways (PERK- eIF2α-ATF4-CHOP, IRE1α-XBP1s and ATF6) (Schroder and Kaufman 2005; Wang et al., 2019a). Notably, although ER stress and UPR are protective mechanism for cells and organisms, excessive ER stress and/or the compromised UPR activate numerous apoptotic signaling pathways rather than restore ER protein-folding homeostasis (Cao 2015). ER stress and UPR have attracted particular interest due to its role in inflammatory responses under pathological conditions (Dandekar et al., 2015; Rahmati et al., 2018). Accumulating findings reveal that ER stress and UPR are involved in the occurrence and development of UC (Kaser and Blumberg 2009; Ma et al., 2017). Unresolved ER stress and/or dysregulated UPR may cause UC by inducing epithelial cell death, impairing intestinal barrier function, and activating inflammatory response in the gut. Therefore, regulation of inordinate ER stress and UPR is considered to be a potential therapeutic approach to treat UC.

Artesunate (ARS), a hemisuccinate derivative of artemisinin (ART) isolated from the herb artemisia annua, is a well established drug for malaria. Besides anti-malarial activity, studies also have demonstrated its medicinal properties like antioxidant, anti-inflammatory, anthelmintic, anti-viral and anti-cancer. It has been proved that ARS and its derivatives, such as ARS and SM934 play protective roles through different pathways in experimental colitis (Yang et al., 2012; Hu et al., 2014; Yan et al., 2018). Our previous study confirmed that ARS ameliorates DSS-induced colitis by protecting intestinal barrier and inhibiting inflammatory response (Yin et al., 2020). Two recent reports showed dihyroartemisinin (DHA) could protect alcoholic fatty liver through inhibiting ER stress (Chen et al., 2018; Chen et al., 2019). Accordingly, it can be assumed that ARS should exert the protective effects on colitis by regulating ER stress in colon tissue. Hereby, in this study, a DSS-induced colitis model was employed to investigate the modulating effects of ARS on ER stress and its subsequent events including the alterations of apoptosis related proteins, mucosal barrier and inflammatory response. In addition, the ER stress inhibitor 4-phenyhlbutyric acid (4-PBA) and inducer 2-Deoxy-d-glucose (2-DG) were also used to verify the potential role of ER stress during this process.

## 2 Materials and Methods

### 2.1 Reagents and Materials

All chemicals were of the highest grade purity available. ARS was obtained from Guiling Pharmaceutical Co., Ltd. (Guangxi, China). Dextran sulfate sodium (DSS, 160,110, MW: 36,000–50,000) was bought from MP Biomedicals (Solon, United States). 4-phenyhlbutyric acid (4-PBA, P21005) and 2-Deoxy-d-glucose (2-DG, D8375) were purchased from Sigma-Aldrich (Kansas, MO, United States). Enhanced chemiluminescence (ECL) kit was purchased from Merck Millipore (Billerica, United States). Bicinchoninic acid (BCA) protein assay kit was bought from Beyotime Institute of Biotechnology (Shanghai, China). RNA isolater Total RNA Extraction Reagent, HiScript II Q Select RT SuperMix for qPCR (+gDNA Wiper) and ChamQTM SYBR^®^ qPCR Master Mix were from Vazyme Biotech Co. Ltd. (Nanjing, China). Mouse ELISA kits were all purchased from Boster Biological Technology Co. Ltd. (Wuhan, China).

The following primary antibodies were used: GRP78 (3,177), PERK (3,192), phospho-PERK (Thr980) (p-PERK, 3,179), eIF2α (5,324), phosphor-eIF2α (Ser51) (*p*-eIF2α, 3,398), ATF4 (11,815), IRE1α (3,294), XBP1s (12,782), CHOP (2,895), NF-κB p65 (8,242), phospho-NF-κB p65 (Ser536) (p-p65, 3,033), IκBα (4,812), phospho-IκBα (Ser32) (*p*-IκBα, 2,859), Bax (2,772), Bcl-2 (3,498), ZO-1 (13,663), *β*-actin (4,970) and GAPDH 5,174) were all from Cell Signaling Technology (Danvers, MA, United States). ATF6 (ab203119), phospho-IRE1α (S724) (p- IRE1α, 48,187), Caspases-12 (ab62484) and claudin-1 (ab242370) were purchased from Abcam (Cambridge, United Kingdom). Muc2 (59,859) was purchased from Santa Cruz Biotechnology (Santa Cruz, CA, United States). The corresponding horseradish peroxidase (HRP)-conjugated secondary antibodies (111–035–003 and 115–035–003) were bought from Jackson Immuno Research (West Grove, PA, United States). secondary antibody which conjugated to-fluorescence (ab150077 and ab150116) was bought from Abcam (Cambridge, United Kingdom).

### 2.2 Animals and Experimental Schedule

Female ICR mice (18–22 g) were obtained from Comparative Medicine Center of Yangzhou University (Jiangsu, China). All animals housed under specific pathogen-free conditions (23 ± 1°C; 50 ± 10% humidity, 12 h light/dark cycle) with free access to standard food and water. Two independent animal experiments were conducted and confirmed to the guidelines from the Animal Care and Use Committee of Yangzhou University. Acute colitis models were induced in mice by drinking 4% (w/v) DSS water solution for seven days and pharmacotherapy was started with the DSS treatment at the same time (Liu et al., 2017). In the first experiment, mice were randomized into four groups (*n* = 8 each group). Mice in control and ARS groups received regular drinking water, whereas mice in DSS and DSS + ARS groups received 4% DSS water solution. In DSS + ARS and ARS groups, mice were treatment with ARS (30 mg/kg, i. p.) once daily for seven days, and other groups were given the same stimulus with vehicle. In the following second experiment, animals were randomly divided into five groups (*n* = 10 per group) as follows: control, DSS, ARS + DSS, ARS+4-PBA + DSS and ARS+2-DG + DSS groups. 4-PBA (500 mg/kg) and/or 2-DG (500 mg/kg) were dissolved in DSS solution and consumed by mice through drinking. During the experiment, Body weight, stool consistency and rectal bleeding were monitored daily for disease activity index (DAI). All animals were injected with 0.1% pentobarbital sodium (50 mg/kg, i. p.) and sacrificed after seven-day experimental period. The colonic tissues were collected, length measured and stored for subsequent analysis.

### 2.3 Evaluation of the Disease Activity Index

The DAI was determined by combining weight loss, scores of bleeding, stool consistency and was based on a scoring system as follows: no body weight loss was scored as 0, 1 point for 1–5% weight loss; 2, 6–10%; 3, 11–20%; and 4, >20%; no blood was scored as 0, 2 points for positive occult blood, and 4 points for gross bleeding; well-formed stool pellets were scored as 0, pasty stools but not adhering to the anus as 2 points, and liquid stools as 4 points. The occult blood was measured by o-toluidine method.

### 2.4 Western Blot Analysis

Proteins from colon tissues were extracted by ice-cold RIPA buffer (APPLYGEN) and quantified with BCA method. After boiled with loading buffer, equal amounts of protein in each sample (50 μg) were prepared for electrophoresis on 8–12% SDS-PAGE, and then electro-blotted onto PVDF membranes (Merck Millipore). The immunoblot was incubated with 5% skim milk for 1 h at room temperature (RT), followed by incubation overnight with primary antibodies at 4°C (all antibodies were diluted following instructions). After three times washes with TBST, the membranes were then incubated with species-specific horseradish peroxidase-conjugated secondary antibodies (diluted 1:5,000) at RT for 1 h. After three times wishes with TBST, protein signal bands were visualized on a Chemidoc XRS (Bio-Rad, Marnes-la-Coquette, France) by an enhanced chemiluminescence kit and qualified using the ImageJ software. Each experiment was performed in triplicate.

### 2.5 Hematoxylin-Eosin Staining

For histopathological analysis, colon segments fixed in PBS containing 4% paraformaldehyde were processed using routine histological methods and embedded in paraffin. The colonic tissue blocks were sliced into 4 μm sections and then stained by hematoxylin and eosin (H&E) for detect the severity of inflammatory cell infiltration, the extent of mucosal injury, and crypt damage. All specimens were photographed under bright-field microscope (Nikon Corporation, Tokyo, Japan).

### 2.6 Real-Time Quantitative Polymerase Chain Reaction

Total RNA from colonic tissue was extracted using Triol isolation reagent and the concentration and purity were measured spectrophotometrically at 260 nm/280 nm. Then, complementary DNA (cDNA) was synthesized from 2 μg of total RNA according to the Prime Script™ RT reagent Kit. Relative mRNA levels of genes were quantified using SYBR Green PCR Master Mix via a CFX connect real-time PCR system (BIO-RAD, US) with target genes primer sequences listed in Table 1. The amplification conditions were as follows: 95°C for 2 min, followed by 40 cycles of 95°C for 15 s and 60°C for 30 s. The expression levels of each target gene were quantified using 2^-△△Ct^ comparative method.

**TABLE 1:** Primers used for real-time PCR in this study.

	Sequence of primers (5′→3′)	Length
IL-1β	R: ATCTCGCAGCAGCACATCA	193
	F: CCAGCAGGTTATCATCATCATCC	
IL-6	R: TTCCATCCAGTTGCCTTCTTG	141
	F: AATTAAGCCTCCGACTTGTGAA	
TNF-α	R: GCAGCCTGTCTCCTTCTATGA	155
	F: TGAAGCAGCAGCCAGCAA	
GAPDH	R: GTTGGATTCTGGGGACGGT	178
	F: TGGTCACGAAGGAATAGCC	

### 2.7 Enzyme-Linked Immunosorbent Assay

Homogenates of colon tissues were extracted by ice-cold RIPA buffer and the concentrations were determined using BCA protein assay kit. The protein levels of IL-1β, IL-6 and TNF-α in colon homogenates were respectively quantified by ELISA kits according to the manufacturer’s protocol, and then detected with a microplate reader at 450 nm absorbance.

### 2.8 Immunohistochemistry and Immunofluorescence Staining

For immunohistochemistry staining, Paraffin-embedded colonic tissues were sectioned at 4 μM and mounted on slides. After dewaxing and rehydration, antigen retrieval was performed by microwaving the sections in 0.01 M sodium citrate buffer (pH 6.0), followed by endogenous peroxidase activity black via incubating with 3% hydrogen peroxide in methanol. Then, Sections were treated with 2% bovine serum albumin (BSA) in PBS at RT for 1 h to block nonspecific binding sites, and incubated with GRP78 and CHOP antibodies (diluted respectively 1:200 and 1:400) overnight at 4°C. Sections were washed and incubated with appropriate HRP-conjugated secondary antibodies for 2 h at room temperature, developed in 3,3′-diaminobenzidine (DAB) for 10 min, counterstained with hematoxylin, and mounted in resin. Slices were photographed under bright-field microscope (Nikon Corporation, Tokyo, Japan).

For immunofluorescence staining, antigen retrieved sections were blocked by 2% BSA at RT for 1 h, followed by respective incubation with claudin-1 and Muc2 antibodies (diluted 1:200) overnight at 4°C. After washing, sections were subsequently incubated with fluorescence-conjugated secondary antibodies (diluted 1:1,000). The nuclei were stained by 4′,6-diamidino-2-phenylindole (DAPI) for 10 min. Finally, sections were photographed using a fluorescence microscope photograph system (Nikon Corporation, Tokyo, Japan). And ImageJ was used to analyze the differences between the tested samples.

### 2.9 Statistical Analyses

All experimental data were presented as mean ± SD of at least three independent experiments. One-way analysis of variance (ANOVA) was utilized to perform statistical analyses followed by the LSD multiple-comparison post hoc test among different groups via IBM SPSS Statistics 22.0 software. Diagrams were undertaken using Graph Pad PRISM (version 5.0) software. *p* < 0.05 was considered to be statistically significant.

## 3 Results

### 3.1 Artesunate Attenuated Endoplasmic Reticulum Stress in Dextran Sulfate Sodium-Challenged Colon Tissues

**FIGURE 1:**
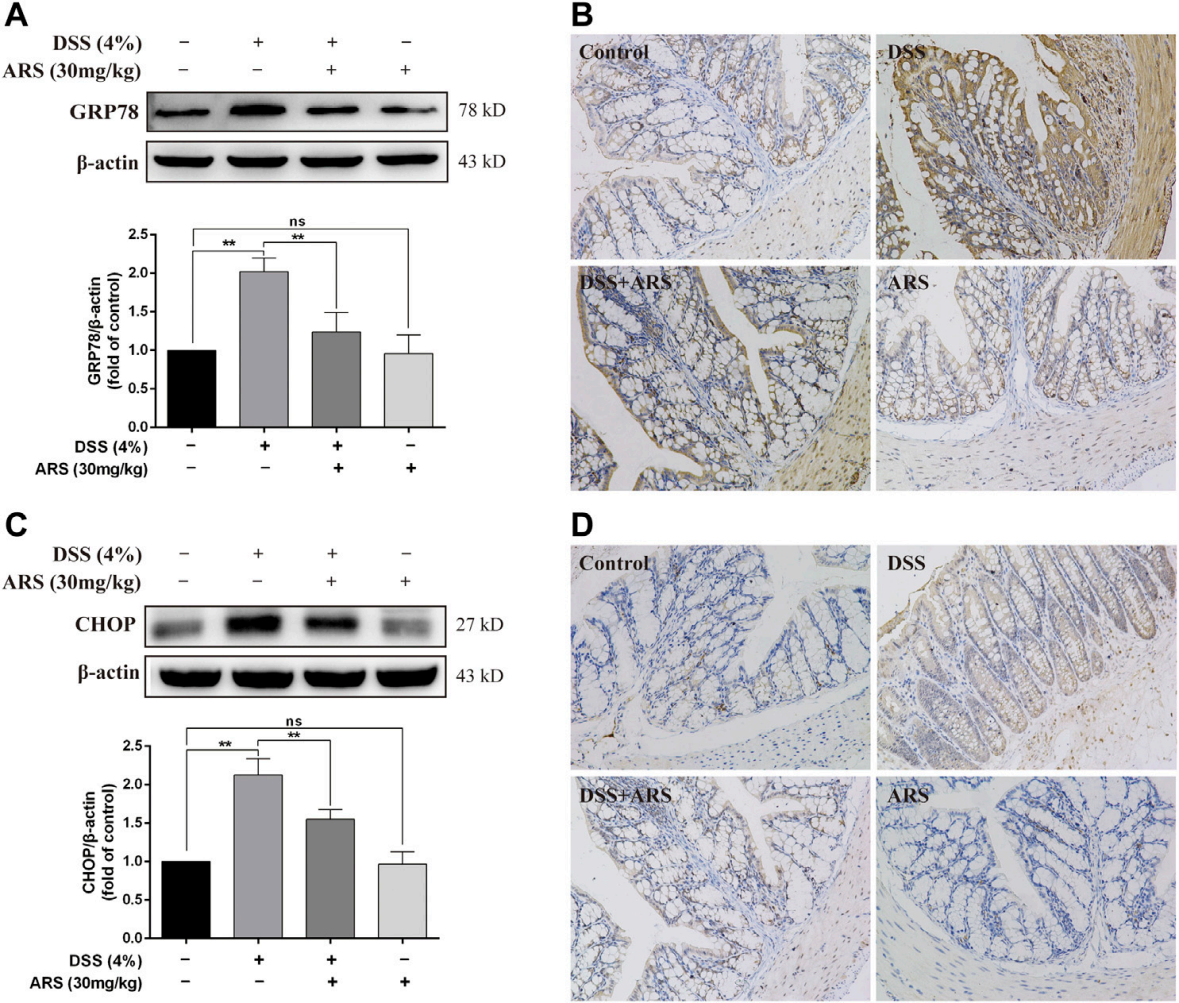
ARS attenuated ER stress in DSS-challenged colon tissues. **(A)** The immunoblot analysis and **(B)** immunohistochemistry test of GRP78 in the colonic tissues from each group. **(C)** The immunoblot analysis and **(D)** immunohistochemistry test of CHOP in colonic tissues. Data are presented as mean ± SD (*n* = 3). *p < 0.05, **p < 0.01.

In order to observe the effect of DSS on ER stress in colon and the role of ARS in regulating ER stress, two marker proteins relevant to ER stress response were examined respectively using western blot and immunohistochemistry assay. Western blot analysis showed the protein levels of glucose-regulated protein (GRP78) (Figure 1A) and C/EBP-homologous protein (CHOP) (Figure 1C) were significantly increased after 7 days DSS exposure (*p* < 0.01). However, the elevation of these two proteins was significantly inhibited by ARS (*p* < 0.01). Meanwhile, the results of immunohistochemistry also suggested that ARS could inhibit the increasing expression of GRP78 and CHOP induced by DSS (Figures 1B,D), indicating ARS alleviated DSS-induced ER stress in colon tissues of mice.

### 3.2 Artesunate Selectively Attenuated Unfolded Protein Response Pathways in Dextran Sulfate Sodium-Challenged Colon Tissues

**FIGURE 2:**
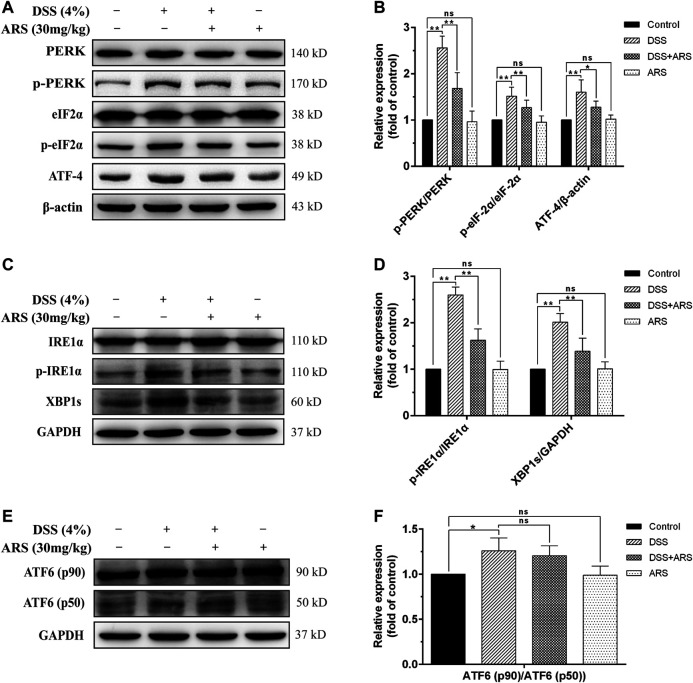
ARS selectively attenuated UPR pathways in DSS-challenged colon tissues. **(A)** The expression of major proteins in PERK-eIF2α-ATF4-CHOP signaling pathway. **(B)** Quantified results from **(A)**. **(C)** The expression of major proteins in IRE1α-XBP1 signaling pathway. **(D)** Quantified results from **(C)**. **(E)** The expression of ATF6 was analysis by western blot. **(F)** Quantified results from **(E)**. Data are presented as mean ± SD (*n* = 3). *p < 0.05, **p < 0.01.

Considering the role of UPR three sensor signaling pathways in response to ER stress, the main marker proteins in these pathways were determined by Western blot assay as shown in Figure 2. As expected, DSS exposure caused a significant increase in the protein ratio of p-PERK/PERK, *p*-eIF2α/eIF2α (Figures 2A,B), p-IRE1α/IRE1α (Figures 2C,D), ATF6 (p50)/ATF6 (p90) (Figures 2E,F) and in the expression of ATF4 (Figures 2A,B) and XBP1s (Figures 2C,D). Whereas, with the exception of ATF6 (p50)/ATF6 (p90), the other protein levels or/and protein ratios mediated by DSS were all significantly inhibited by ARS treatment. These results, along with the result of CHOP in Figures 1C,D, showed that ARS inhibited DSS-induced ER stress in mice colon tissues via suppressing the activation of PERK-eIF2α-ATF4-CHOP and IRE1α-XBP1 signaling pathways.

### 3.3 Effect of 4-PBA and 2-DG on the Inhibitory Role of Artesunate in Dextran Sulfate Sodium-Induced Endoplasmic Reticulum Stress

**FIGURE 3:**
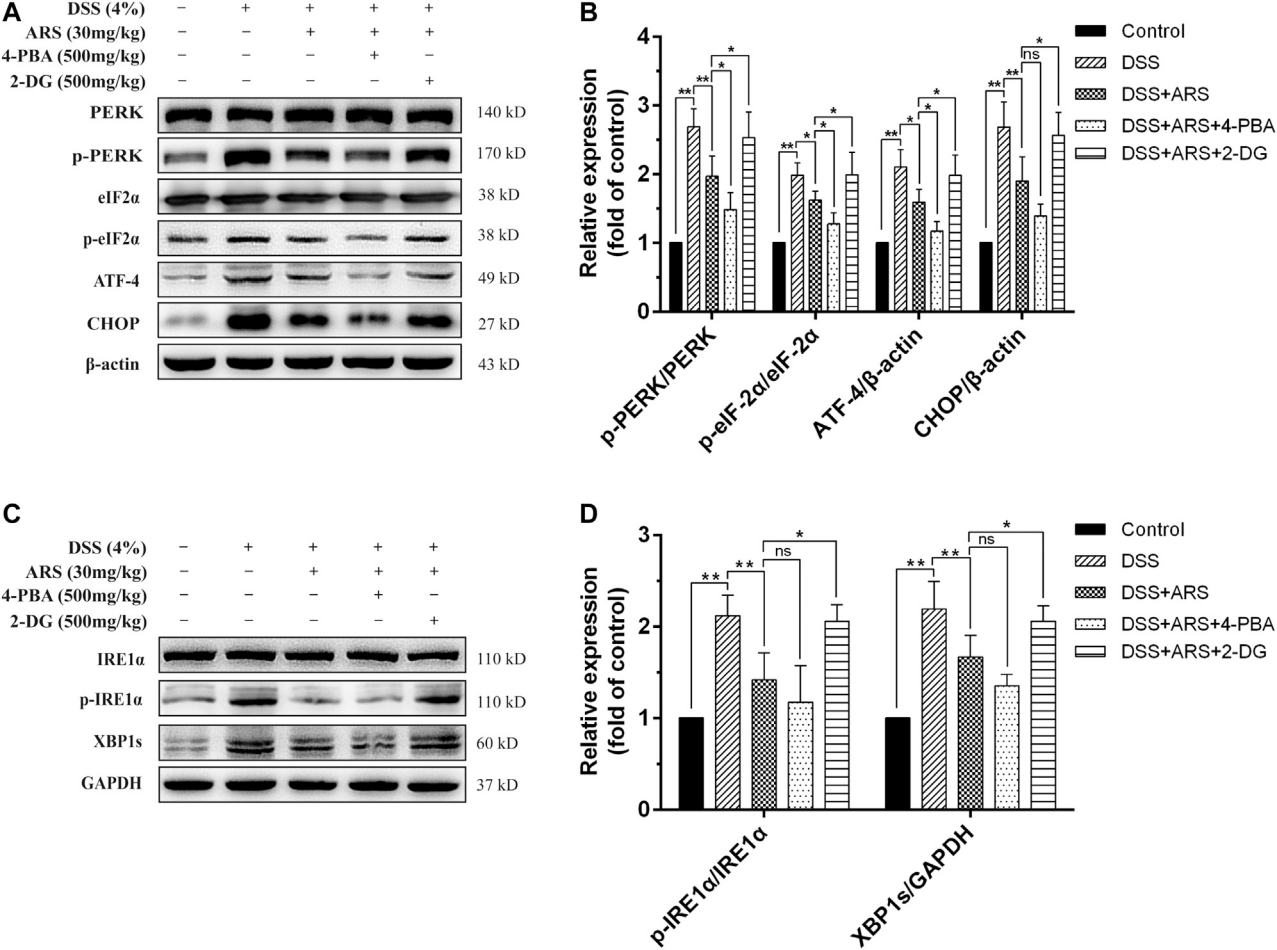
Effect of 4-PBA and 2-DG on the inhibitory role of ARS in DSS-induced ER stress. **(A)** The immunoblot analysis of major proteins in PERK-eIF2α-ATF4-CHOP signaling pathway. **(B)** Quantified results from **(A)**. **(C)** The expression levels of major proteins in IRE1α-XBP1 signaling pathway were assessed by western blot. **(D)** Quantified results from **(C)**. Data are presented as mean ± SD (*n* = 3). *p < 0.05, **p < 0.01.

In the following second experiment, we firstly confirmed the regulatory effects of 4-PBA (an ER stress inhibitor) and 2-DG (an ER stress inducer) on PERK-eIF2α-ATF4-CHOP and IRE1α-XBP1 pathways based on ARS treatment in mice colonic tissues during DSS exposure. Similar to the results of Figure 2, ARS treatment significantly suppressed the activation of PERK- eIF2α-ATF4-CHOP and IRE1α-XBP1s signaling pathways. On the basis of ARS processing, 4-PBA co-treatment further markedly downregulated the protein ratio of p-PERK/PERK, *p*-eIF2α/eIF2α and ATF4 expression. (Figures 3A,B). Although the ratio of p-IRE1α/IRE1α as well as the expression of XBP1s (Figures 3C,D) and CHOP (Figures 3C,D) were revised down by co-treatment with 4-PBA, no remarkable difference was observed. In contrast, 2-DG co-treatment markedly abrogated the inhibitory effects of ARS on PERK-eIF2α-ATF4-CHOP and IRE1α-XBP1 signaling pathways.

### 3.4 4-PBA and 2-DG Affect the Effectiveness of Artesunate on Endoplasmic Reticulum Stress-Related Apoptosis

**FIGURE 4:**
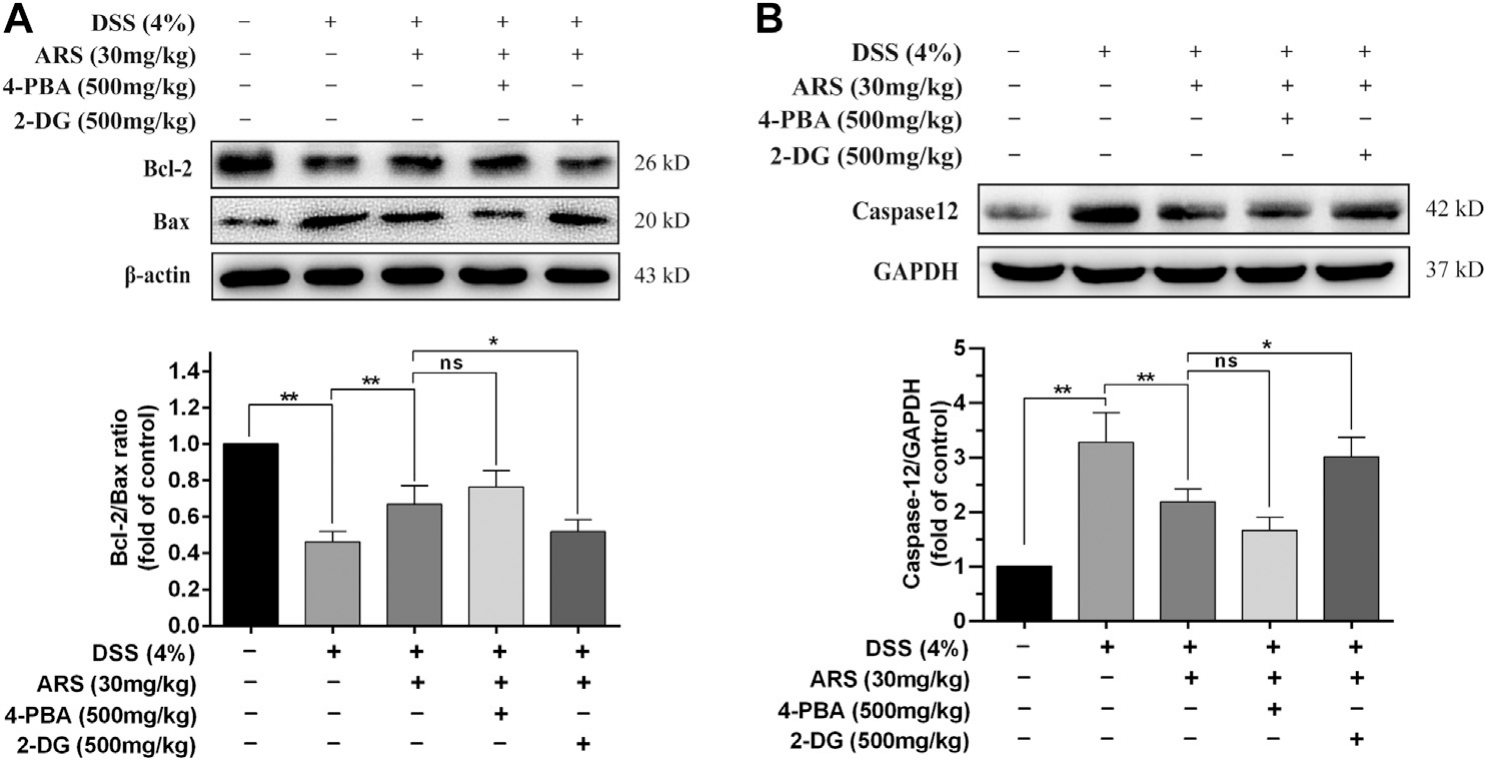
4-PBA and 2-DG affect the effectiveness of ARS on ER stress-related apoptosis. **(A)** Immunoblot analysis of Bcl-2/Bax ratio. **(B)** The expression of caspases12 was analysis by western blot. Data are presented as mean ± SD (*n* = 3). *p < 0.05, **p < 0.01.

If ER function cannot be restored, the prolonged activation of UPR could cause ER stress-related apoptosis via up-regulating CHOP, which induces apoptosis through mitochondria-dependent pathway (Hu et al., 2018). Therefore, the expression of proto-oncoproteins (Bcl-2 and Bax) in colon tissues were detected. Concurring with the result of CHOP, DSS exposure induced significant downregulation of Bcl-2/Bax ratio, which was remarkably abrogated by ARS treatment (Figure 4A). Although co-treatment of 4-PBA did not further significantly promote the upregulation of Bcl-2/Bax ratio, 2-DG markedly abolished the role of ARS. Furthermore, one important ER stress-related apoptotic protein, caspases12, was detected. As shown in Figure 4B, the expression of caspases12 was remarkably increased under the challenge of DSS. However, ARS treatment significantly reduced the expression of caspases12. Similarly, 4-PBA did not further significant downregulating the expression of caspases12, and 2-DG markedly abolished the inhibitory effect of ARS.

### 3.5 Endoplasmic Reticulum Stress Inhibition Involves in the Protective Effect of Artesunate on Clinical and Histological Symptoms in Dextran Sulfate Sodium-Induced Colitis

**FIGURE 5:**
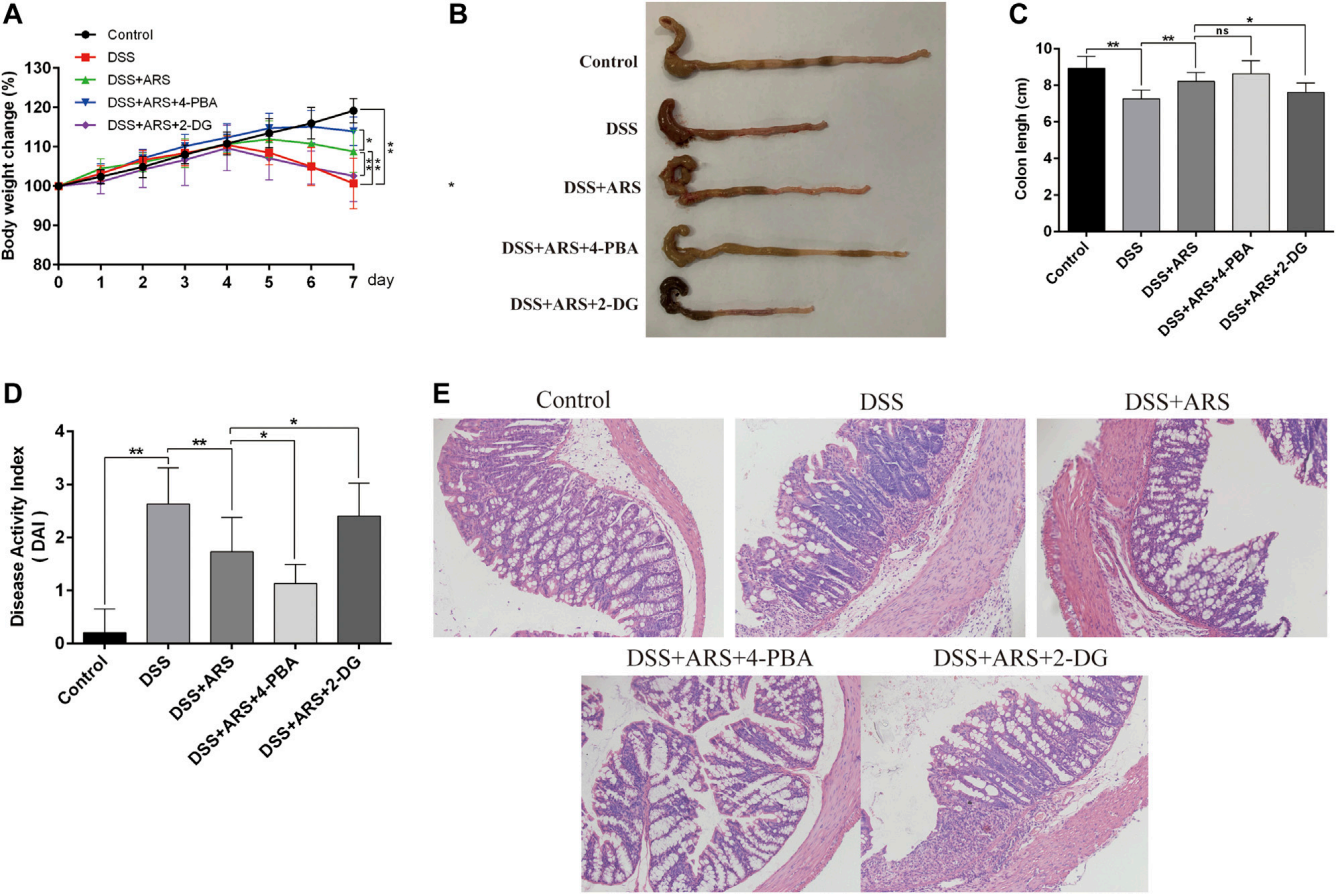
ER stress inhibition involves in the therapeutic effect of ARS on clinical and histological symptoms in DSS-induced colitis. ER stress inhibitor 4-PBA and inducer 2-DG were used to verify the role of ER stress on the therapeutic effect of ARS. **(A)** The body weight change of mice in each group. **(B)** Macroscopic appearances of colon in different groups. **(C)** The average colon length of mice in each group. **(D)** Disease activity index of colon, a composite measure of weight loss, stool, consistency and blood in stool. Data are presented as mean ± SD (*n* = 10). *p < 0.05 and **p < 0.01. **(E)** The pathological evaluation of colonic tissues was performed using H.E. staining and observed under 200× phase contrast microscope.

The clinical symptoms were determined by changes of body weight, colon length and DAI scores, and the pathological changes were measured by observing the colon sections via HE staining. As shown in Figures 5A–D, DSS challenge markedly reduced body weight, colon length and increased DAI scores (*p* < 0.01). However, ARS administration notably alleviated DSS effects (*p* < 0.01). Additionally, in the histological examination, ARS significantly suppressed integrity loss, goblet cell damage and inflammatory cell infiltration induced by DSS in colon tissues (Figure 5E). Furthermore, the results of Figure 5 indicated that comparing with the single treatment of ARS, the co-treatment with 4-PBA further mitigated DSS effects including body weight loss and DAI scores increasing (*p* < 0.05), as well as histological destruction of colon tissues. But, the improvement effect of ARS on clinical symptoms and colonic histology markedly abrogated by co-treatment of 2-DG (*p* < 0.05). Combined with the above results from Figure 3, these data demonstrated that the inhibitory effect of ARS on ER stress plays an important role in protecting DSS-induced colitis.

### 3.6 Endoplasmic Reticulum Stress Inhibition Involves in the Anti-inflammatory Effect of Artesunate on Dextran Sulfate Sodium-Induced Colitis

**FIGURE 6:**
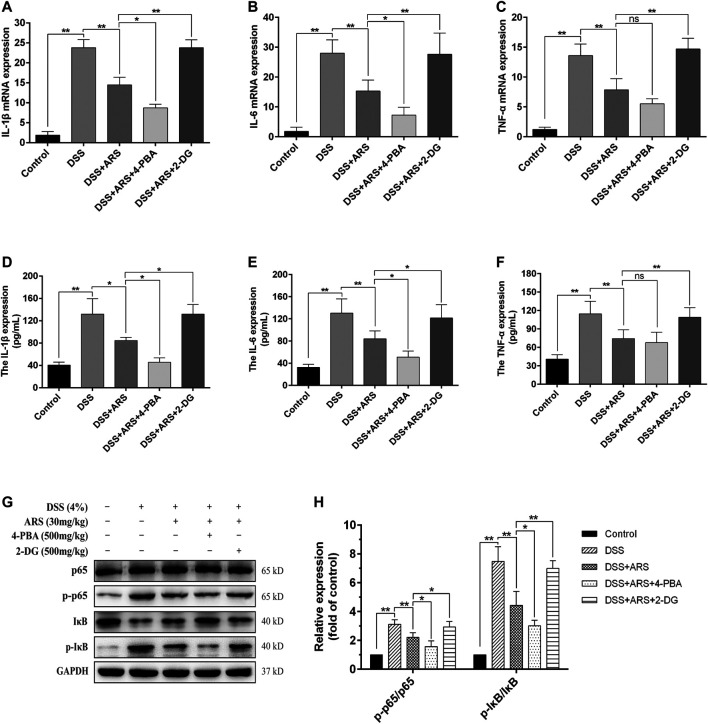
ER stress inhibition involves in the anti-inflammatory effect of ARS on DSS-induced colitis. 4-PBA and 2-DG were respectively used as ER stress inhibitor and inducer to demonstrate the role of ER stress on the anti-inflammatory effect of ARS. **(A–C)** The mRNA expressions of cytokines IL-1β, IL-6, and TNF-α were detected by RT-qPCR. **(D–F)** The protein expressions of cytokines IL-1β, IL-6, and TNF-α were detected by ELISA. **(G)** Immunoblot analysis of the expression levels of p65/p-p65 and *p*-IκB/IκB ratio. **(H)** Quantified results from **(G)**. All the values were expressed as mean ± SD (*n* = 3), *p < 0.05, **p < 0.01.

To verify whether the anti-inflammatory effect of ARS is related to the suppression of ER stress, the mRNA expression of pro-inflammatory factors and the activation of NF-κB were detected with the help of 4-PBA and 2-DG. The results showed that ARS treatment notably downregulated the mRNA (Figures 6A–C) and protein (Figures 6D–F) expression of IL-1β, IL-6, TNF-α, and the protein ratio of p-p65/p65 and *p*-IκBα/IκBα (Figures 6G,H) in colon tissues in colitis mice (*p* < 0.01). Moreover, ARS-depressed levels of IL-1β, IL-6 (not TNF-α) and protein ratio of p-p65/p65 and *p*-IκBα/IκBα were further significantly down-regulated by co-treatment with 4-PBA. Conversely, the depression effects of ARS were markedly abrogated by co-treatment with 2-DG. These data collectively suggested that the inhibitory effect of ARS on ER stress plays an important role in modulating the expression of pro-inflammatory factors and the activation of NF-κB signaling pathway in DSS-induced colitis.

### 3.7 The Inhibitory Effect of Artesunate on Endoplasmic Reticulum Stress Involves in Maintaining Intestinal Barrier Integrity

**FIGURE 7:**
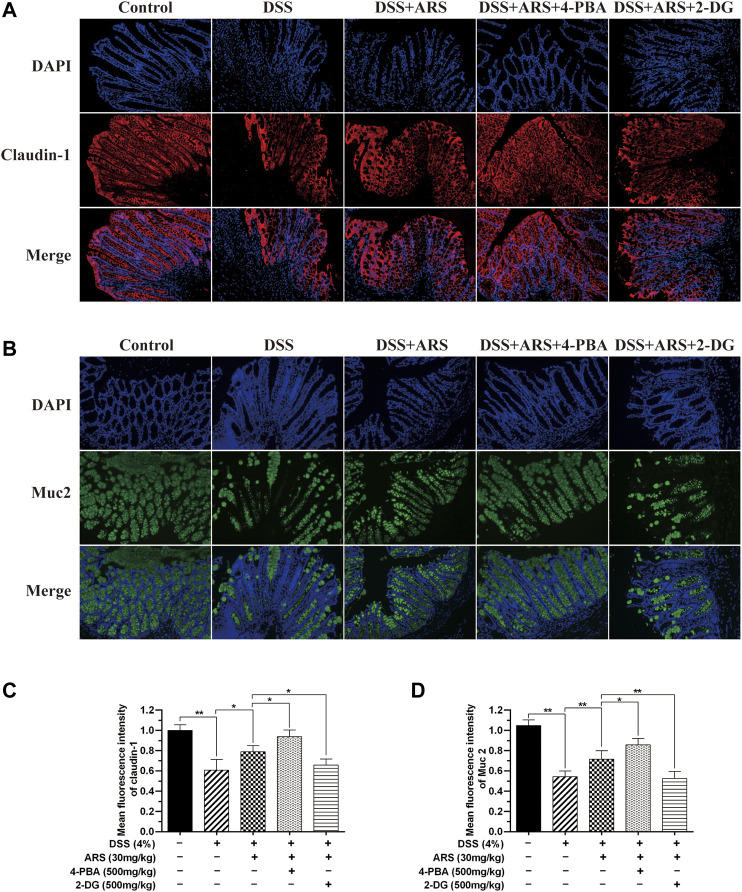
The inhibitory effect of ARS on ER stress involves in maintaining intestinal barrier integrity. Sections of colonic tissues were immunostained with DAPI and antibodies and then observed under 200× fluorescence microscope. The red fluorescence represents the amount of claudin-1 **(A)**, The green fluorescence represents the amount of Muc2 **(B)**. The blue fluorescence is the nucleus stained by DAPI. **(C)** Quantified results from **(A)**. **(D)** Quantified results from **(B)**. Data are presented as mean ± SD (*n* = 3). *p < 0.05, **p < 0.01.

The intestinal barrier is the first-line defense against challenge. In this study, mucin protein Muc2 and tight junction protein, claudin-1 in mice colon were detected by immunofluorescence staining. Same as our previous study (Yin et al., 2020), DSS stimulation significantly reduced the expression of Muc2 and claudin-1 compared with control group, and ARS treatment notably inhibited the reduction of Muc2 and claudin-1. Additionally, the results in Figure 7 demonstrated that ARS-elevated protein levels of Muc2 and claudin-1 were markedly up-regulated by co-treatment with 4-PBA. On the contrary, ARS-upregulated protein levels of Muc2 and claudin-1 were remarkably depressed by co-treatment with 2-DG.

## 4 Discussion

Due to the increasing prevalence and incidence, UC has become a heavy societal burden (Yan et al., 2018). Many factors can induce UC, so finding out common pathogenic mechanisms and effective drugs to treat these pathogenic mechanisms are urgently needed. Studies have shown that incurable ER stress and UPR activation are significant mechanisms of intestinal inflammation in epithelial tissues and are considered as potential therapeutic targets (Ma et al., 2017; Takagi et al., 2019). DSS-induced mouse colitis is deemed a well-characterized experimental colitis model for identifying molecular mechanisms involving in the progression of UC, and investigating the curative effect of drugs on colitis (Zhu et al., 2017). The present study aimed to investigate underlying mechanisms of ARS in alleviating DSS-induced colitis in mice. We found that ARS markedly relieved excessive ER stress via suppressing the activation of PERK- eIF2α-ATF4-CHOP and IRE1α-XBP1s signaling pathways, and the inhibitory effect of ARS on excessive ER stress participated in anti-inflammatory response and maintaining intestinal barrier integrity.

Numerous studies have shown that ER stress is involved in the pathology of UC, and inhibiting excessive ER stress could play a better therapeutic role in preventing colitis (Kaser and Blumberg 2009; Liu et al., 2017). As a consequence of ER stress, the activation degree of UPR signaling pathway determines cells will re-establish homeostasis or initiate inflammation or even activate cell death programs, and the regulation program is complicated and has been reviewed in previous reports by other researchers (Kaser and Blumberg 2009; Kaser et al., 2011; Cao 2015; Ma et al., 2017). Severe or prolonged ER stress and uncontrollable UPR can activate ER stress-associated cell death signaling (Tabas and Ron, 2011; Hetz, 2012). Studies point out that CHOP and caspases 12 are critical mediators on ER stress-induced apoptosis (Kitamura 2011; Ma et al., 2016). Additionally, ER stress could induce mitochondrial-dependent cell death by regulating B cell lymphoma 2 (Bcl-2) protein family, which might be the vital mechanism of CHOP inducing apoptosis (Hetz 2012). In the present study, DSS exposure caused high expression of GRP78, activation of all the three signaling pathways of UPR, and consequential high expression of caspases12 and low ratio of Bcl-2/Bax. Whereas, ARS treatment significantly inhibited GRP78 expression as well as PERK- eIF2α-ATF4-CHOP and IRE1α-XBP1s signaling pathways activation. Furthermore, ARS relieved the DSS-induced apoptosis. These results demonstrated that ARS alleviates excessive ER stress challenged by DSS via inhibiting above-mentioned two signaling pathways. What is noteworthy is that the three signaling branches of UPR have different functions and are not necessarily activated simultaneously (Bogaert et al., 2011), and the role of PERK- eIF2α-ATF4-CHOP and IRE1α-XBP1s signaling pathways receive more attention in UC. Combining with the above results, it portended that ARS could well relieve DSS-induced colitis via suppressing excessive ER stress. Also, in this study, the classic ER stress inhibitor 4-PBA and inducer 2-DG were proved could be used as tools to observe the effect of ARS on ER stress in resisting colitis in the following research.

UC is characterized by numerous clinical manifestations, including abdominal pain, diarrhea, blood in the stool and weight loss (Fatani et al., 2016; Feuerstein et al., 2019). In DSS-induced experimental colitis, the changes of these clinical symptoms and histological lesions are often used as indicators of drug efficacy (Yan et al., 2018; Wang et al., 2019b). In this study, ARS markedly improved clinical and histopathological symptoms, which is similar with our previous report (Yin et al., 2020). Moreover, with the help of 4-PBA and 2-DG, the inhibitory effect of ARS on PERK- eIF2α-ATF4-CHOP and IRE1α-XBP1s signaling pathways activation was proved to play an important role in improving clinical and histopathological symptoms. The results are similar to previous studies that alleviating excessive ER stress can improve symptoms of colitis and colon lesions (Hasnain et al., 2013; Kim et al., 2019).

Being important inflammatory mediators, cytokines participate in many pathophysiologic processes (Neurath 2014; Zhao et al., 2015). It has been demonstrated that various cytokines, such as IL-1β, IL-6 and TNF-α, contribute to the initiation and development of UC (Neurath 2014). Nuclear transcription factor NF-κB generally regulates the production of pro-inflammatory cytokines in inflammatory process and has been verified playing a key role in the pathogenesis of colitis (Choi et al., 2017). In addition, activation of NF-κB has been reported to be a consequence of ER stress (Arvin et al., 2012). In our study, ARS treatment markedly inhibited the expression of IL-1β, IL-6 and TNF-α as well as the activation of NF-κB pathway evidenced by downregulating the protein ratio of p-p65/p65 and *p*-IκBα/IκBα. Furthermore, the inhibitory effects of ARS on inflammatory cytokines and NF-κB pathway were further improved by co-treatment with 4-PBA and abrogated by co-treatment with 2-DG. Combining with the inhibitory effect of ARS on PERK- eIF2α-ATF4-CHOP and IRE1α-XBP1s signaling pathways, we speculated that the inhibitory effect of ARS on ER stress plays an important role in its anti-inflammatory effect on DSS-induced colitis. In support of our findings, several studies confirmed that under ER stress, PERK-induced phosphorylation of eIF2α activates NF-κB by phosphorylation and consequent degradation of IκBα (Deng et al., 2004), and IRE1α induces activation of NF-κB via repressing IκB translation (Hu et al., 2006). Moreover, one report suggested that XBP1 activation could drive pro-inflammatory transcription in macrophages (Martinon et al., 2010).

**FIGURE 8:**
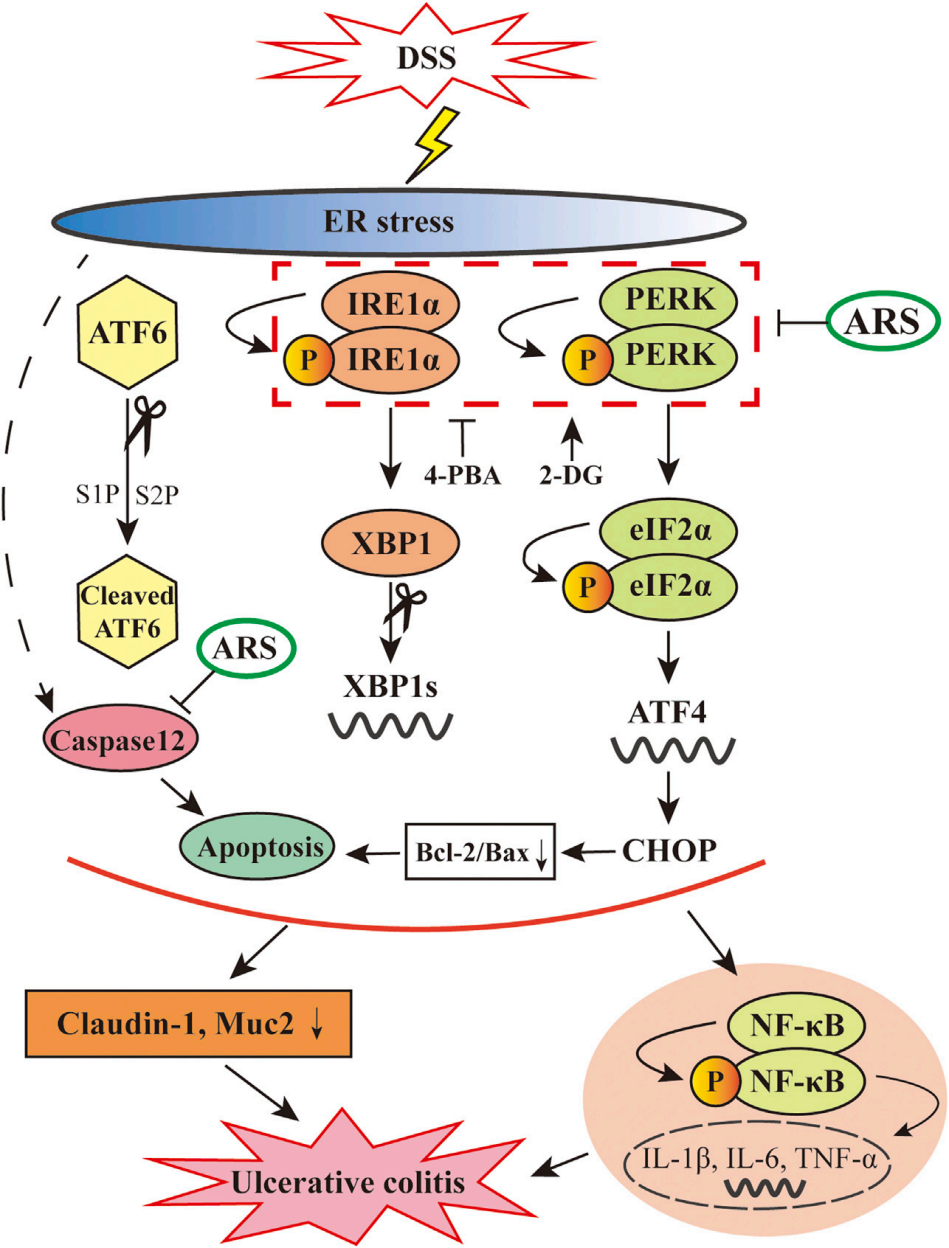
Diagram showing the proposed pathways suppressed by ARS in the colonic tissues of murine UC model induced by DSS. ARS significantly inhibited DSS induced excessive ER stress via suppressing PERK-eIF2α-ATF4-CHOP and IRE1α-XBP1 signaling pathways with the consequent ER stress-associated apoptosis. The relieved effect of ARS on excessive ER stress plays critical role in downregulating the activation of NF-κB and the mRNA expression levels of pro-inflammatory cytokines as well as maintained the expression of claudin-1 and Muc2.

The epithelial barrier is made up of a single layer of epithelial cells, which were tied by tight junction (TJ) proteins. Goblet cells are scattered within the intestinal epithelia, which produce and secrete Muc2 mucin to form the mucus blanket overlying the surface of the intestinal lumen (Zong et al., 2020). TJs and mucus are two important components of the intestinal barrier, which play crucial role in protecting intestinal health respectively via regulating intestinal integrity and permeability, and preventing harmful substances reaching the surface (Merga et al., 2014; Cao et al., 2018; Zong et al., 2020). In the present study, Muc2 and claudin-1 were chosen to be detected, and DSS stimulation considerably reduced the expression of Muc2 and claudin-1, which was markedly inhibited by ARS treatment. Furthermore, ARS-elevated protein levels of Muc2 and claudin-1 were remarkably up-regulated by co-treatment with 4-PBA and depressed by co-treatment with 2-DG. Above results demonstrated that the inhibitory effect of ARS on ER stress is essential to maintaining intestinal barrier integrity in DSS-induced colitis. As evidences, studies in mice demonstrated that ER stress causes reduction in Muc2 biosynthesis due to the translational block induced by the UPR, which decreased the amount of secreted Muc2 (Cornick et al., 2015). As well, one report also verified that ER stress is associated with the disruption of TJ in high-fat feeding mice (Huang et al., 2020). It is noteworthy that reports from Akiyama et al. indicate that PERK-eIF2α-ATF4-induced CHOP expression, rather than IRE1a-XBP1 signaling may be a crucial mediator of epithelial injury (Akiyama et al., 2016). However, how does ARS regulate claudin-1 and Muc2 via mediating PERK-eIF2α-ATF4-CHOP and IRE1α-XBP1 signaling pathways need to be further investigated.

n summary, as depicted in Figure 8, the data showed that ARS treatment significantly relieves excessive ER stress challenged by DSS via inhibiting the activation of PERK-eIF2α-ATF4-CHOP and IRE1α-XBP1 signaling pathways and the subsequent ER stress-associated apoptosis. Moreover, the alleviative role of ARS on excessive ER stress plays a crucial role in inhibiting inflammatory response and protecting intestinal barrier in colitis mice. Above-mentioned effects of ARS improved the clinical and histopathological symptoms of colitis. The study demonstrated that suppressing excessive ER stress might be the main protective mechanism of ARS against experimental colitis.

## Data Availability

The original contributions presented in the study are included in the article/Supplementary Material, further inquiries can be directed to the corresponding author.
